# Evolving indications and long‐term oncological outcomes of risk‐reducing bilateral nipple‐sparing mastectomy

**DOI:** 10.1002/bjs5.50117

**Published:** 2018-11-26

**Authors:** S. R. Grobmyer, H. J. Pederson, S. A. Valente, Z. Al‐Hilli, D. Radford, R. Djohan, R. Yetman, C. Eng, J. P. Crowe

**Affiliations:** ^1^ Breast Services Division, Department of General Surgery, Cleveland Clinic Cleveland Ohio USA; ^2^ Department of Plastic Surgery, Cleveland Clinic Cleveland Ohio USA; ^3^ Genomics Medicine Institute, Cleveland Clinic Cleveland Ohio USA

## Abstract

**Background:**

Bilateral nipple‐sparing mastectomy (NSM) is a technically feasible operation and is associated with excellent cosmetic outcomes. The aim of this study was to evaluate trends in patient characteristics, indications for surgery and long‐term outcomes of bilateral NSM for breast cancer risk reduction over time.

**Methods:**

A review of a single‐centre experience with bilateral NSM performed between 2001 and 2017 for breast cancer risk reduction in patients without breast cancer was performed. Trends in patient characteristics and indications for surgery were evaluated over four time intervals: 2001–2005, 2006–2009, 2010–2013 and 2014–2017. Statistical analysis was performed using χ^2^ tests.

**Results:**

Over the study period, 272 NSMs were performed in 136 patients; their median age was 41 years. The number of bilateral NSMs performed increased over time. The most common indication was a mutation in breast cancer‐associated genes (104 patients, 76·5 per cent), which included *BRCA1* (62 patients), *BRCA2* (35), *PTEN* (2), *TP53* (3) and *ATM* (2). Other indications were family history of breast cancer (19 patients, 14·0 per cent), lobular carcinoma *in situ* (10, 7·4 per cent) and a history of mantle irradiation (3, 2·2 per cent). The proportion of patients having a bilateral NSM for mutation in a breast cancer‐associated gene increased over time (2001–2005: 2 of 12; 2006–2009: 9 of 17; 2010–2013: 34 of 41; 2014–2017: 61 of 66; *P* < 0·001). Mean follow‐up was 53 months; no breast cancers were found during follow‐up.

**Conclusion:**

The use of bilateral NSM for breast cancer risk reduction is increasing and the indications have evolved over the past 16 years. These excellent long‐term oncological results suggest that bilateral NSM is a good option for surgical breast cancer risk reduction.

## Introduction

Risk‐reducing simple mastectomy, subcutaneous mastectomy and skin‐sparing mastectomy have all been demonstrated to be associated with breast cancer risk reduction in patients with a strong family history of breast cancer and in patients with mutations in *BRCA1* and *BRCA2* genes^1^, ^2^, ^3^. Over the past 15 years, nipple‐sparing mastectomy (NSM) has emerged as an option for the treatment and prevention of breast cancer in selected patients^4^ ^5^. These reports and others^6^, ^7^, ^8^, ^9^, ^10^, ^11^, ^12^, ^13^, ^14^ have established the technical feasibility of NSM. Most reports of NSM have focused on patients with a diagnosis of breast cancer, not NSM performed for breast cancer risk reduction in high‐risk patients; in one previous series^5^ only 13 of 111 patients had bilateral NSM for breast cancer risk reduction. There have been several reports^9^ ^11^, ^15^ ^16^ of bilateral NSM for breast cancer risk reduction, but these focused only on patients with mutations in *BRCA1* and *BRCA2*. In addition, many of these series report only short‐term oncological follow‐up. The limited published experience, relatively short‐term reported follow‐up and presence of terminal ductal lobular units in the nipple–areolar complex have led some groups^17^, ^18^, ^19^, ^20^ to question the long‐term oncological safety of NSM for risk reduction in patients with an increased risk of breast cancer.

Since 2001, bilateral NSM for breast cancer risk reduction in high‐risk patients with a genetic predisposition to breast cancer, a history of high‐risk or atypical breast lesions, a strong family history of breast cancer, and history of mantle irradiation has been offered at Cleveland Clinic. The aims of this study were to determine whether the performance of risk‐reducing NSM is increasing over time and whether the indications for risk‐reducing bilateral NSM have changed over the past 17 years in the context of the increasing incorporation of more extensive germline genomic testing in clinical practice. It also aimed to ascertain whether bilateral NSM for risk reduction is oncologically safe and associated with low rates of subsequent breast cancer in high‐risk patients with long‐term follow‐up.

## Methods

Approval for this study was obtained by the Cleveland Clinic Institutional Review Board. Local databases were reviewed to identify patients who underwent risk‐reducing bilateral NSM at Cleveland Clinic between 2001 and 2017. Patients found to have occult cancer at the time of surgery, those who underwent contralateral risk‐reducing mastectomy, and patients with breast cancer or a history of breast cancer were excluded from the analysis. The decision to perform a risk‐reducing bilateral NSM was made by the patient and surgeon. Technical aspects of the procedures have been described previously^4^ ^5^. The absence of cancer in the bilateral mastectomy specimens was confirmed by routine histological analysis. Follow‐up was from the date of surgery to the date of last clinical follow‐up at Cleveland Clinic.

The electronic medical record was reviewed to confirm and update data relevant to the study. Temporal trends were evaluated over four time intervals (2001–2005, 2006–2009, 2010–2013 and 2014–2017) to facilitate analysis of the data over time. The short‐term technical outcomes of bilateral NSM have been well documented^4^ ^5^, ^7^ ^8^, ^14^ and are beyond the scope of this report.

### Statistical analysis

Statistical analysis was performed with the χ^2^ test using StatView® 4 (SAS Institute, Cary, North Carolina, USA). *P* < 0·050 was considered statistically significant.

## Results

**Figure 1:**
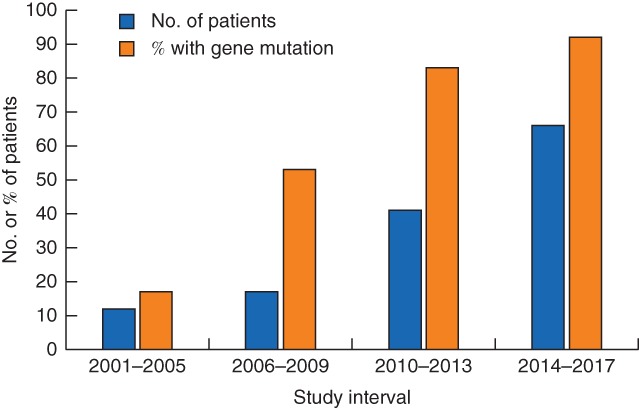
Trends over time in use of risk‐reducing bilateral nipple‐sparing mastectomy and proportion of patients with pathogenic mutation in breast cancer genes

A total of 136 patients (135 women and 1 man) underwent risk‐reducing bilateral NSM between October 2001 and May 2017. Their median age was 41 (range 20–67) years. The number of patients having risk‐reducing bilateral NSM increased over the study interval (*Fig*. 1).

The most common indication for risk‐reducing bilateral NSM for patients in this series was mutation in a breast cancer‐associated gene (104 patients, 76·5 per cent): *BRCA1*, 62 (45·6 per cent); *BRCA2*, 35 (25·7 per cent); *PTEN,* two (1·5 per cent); *TP53,* three (2·2 per cent); and *ATM,* two (1·5 per cent) (*Table* 1). Other indications were a strong family history of breast cancer (19, 14·0 per cent), lobular carcinoma *in situ*/atypia (10, 7·4 per cent) and a history of mantle field irradiation (3, 2·2 per cent). The proportion of patients who had a risk‐reducing bilateral NSM for mutation in a breast cancer‐associated gene increased significantly over the study interval (2001–2005: 2 of 12 patients; 2006–2009: 9 of 17; 2010–2013: 34 of 41; 2014–2017: 61 of 66) (*P* < 0·001) (*Fig*. 1). A risk‐reducing bilateral NSM for patients with genetic mutations was first performed for *BRCA, PTEN, TP53* and *ATM* in 2004, 2013, 2013 and 2015 respectively.

**Table 1:** Reported series of risk‐reducing bilateral nipple‐sparing mastectomy

Reference	Institutions	Year	No. of risk‐reducing NSMs	Median patient age (years)	Breast cancer risk category (%)	Follow‐up (months)		New cancer (%)
						Mean	Median	
Sacchini *et al*.^7^	MSKCC	2006	84	45	n.d.	n.a.	25	2
	Sao Paulo University							
	European Oncology Institute							
	University of Padua							
Crowe *et al*.^5^	Cleveland Clinic (Cleveland)	2008	26	43	n.d.	n.a.	n.a.	n.a.
Peled *et al*.^11^	UCSF	2014	52	41	*BRCA1* 54	37	n.a.	0
					*BRCA2* 46			
Yao *et al*.^9^	Northwestern University	2015	298	41	*BRCA1* 62	33	n.a.	0·6
	Massachusetts General Hospital				*BRCA2* 46			
Manning *et al*.^15^	MSKCC*	2015	126	39	*BRCA1* 63	n.a.	26	0
					*BRCA2* 29			
					*BRCA* VUS 8			
Moo *et al*.^10^	New York Hospital – Cornell	2016	90	42	*BRCA1/2* 42	n.a.	32	n.a.
					Other 58			
Jakub *et al*.^16^	Mayo Clinic (Rochester, Phoenix, Jacksonville)	2018	404	41	*BRCA1* 58	56	34	0
					*BRCA2* 42			
	UCSF							
	Duke University							
	Moffitt Cancer Center							
	MSKCC							
	University of Pennsylvania							
	Georgetown University							
Grobmyer *et al*. (present series)	Cleveland Clinic (Cleveland)	2018	272	40	*BRCA1* 45.6	53	38	0
					*BRCA2* 25.7			
					*PTEN* 1.5			
					*TP53* 2.2			
					*ATM* 1.5			
					Family history 14·0			
					LCIS/atypia 7·4			
					History of mantle irradiation 2·2			

NSM, nipple‐sparing mastectomy; MSKCC, Memorial Sloan Kettering Cancer Center; n.d., not defined; n.a., not available; UCSF, University of California, San Francisco; VUS, variant of unknown significance; LCIS, lobular carcinoma *in situ*.

Mean and median duration of follow‐up for all patients in the series was 53 and 38 (range 0·5–326) months respectively; 61 patients had follow‐up for more than 4 years. Follow‐up for patients in the series with mutations in breast cancer predisposition genes (*BRCA1, BRCA2, PTEN* and *TP53*) are shown in *Table* 2. No patient undergoing risk‐reducing bilateral NSM in this series developed breast cancer during follow‐up.

**Table 2:** Characteristics of patients with genetic syndromes undergoing risk‐reducing bilateral nipple‐sparing mastectomy

Mutation	No. of patients	Age at bilateral NSM (years)*	Follow‐up (months)		Breast cancer during follow‐up
			Mean	Median	
*BRCA1* or *BRCA2*	97	39 (20–67)	30	42	0
*PTEN*	2	30 (25–35)	54	54	0
*TP53*	3	29 (20–40)	26	32	0
*ATM* †	2	48·5 (47–50)	30	30	0

* Values are median (range).

† Patients also had a significant family history of breast cancer. NSM, nipple‐sparing mastectomy.

## Discussion

NSM has the advantage of preserving the nipple and skin envelope to optimize cosmesis following mastectomy, and it facilitates the reconstruction process^7^ ^21^. Patient satisfaction with the appearance of the nipple–areolar complex is high after NSM, and the majority of patients are satisfied with their decision to undergo NSM^21^. It has been shown^22^ that NSM is associated with higher patient psychosocial and sexual well‐being compared with that in patients having skin‐sparing mastectomy with removal of the nipple–areolar complex. Others^23^ have found no difference in satisfaction with overall outcome between patients having NSM and skin‐sparing mastectomy with reconstruction. It is noteworthy that not all patients are optimal candidates for risk‐reducing NSM, particularly those with significant ptosis, very large breast size or high BMI, and those who are active smokers.

The present large single‐centre experience of NSM for breast cancer risk reduction reinforces the oncological safety of this procedure, as no breast cancers developed among patients in this series. In a series of 63 patients of similar age with *BRCA* mutations undergoing surveillance, 12 per cent had developed breast cancer at a mean follow‐up of 2·9 years^3^. Other series that have documented breast cancer risk reduction associated with bilateral NSM in patients with *BRCA* mutations are summarized in *Table* 1. The present series documents that the procedure has been used increasingly over time for breast cancer risk reduction, similar to the recent report of Jakub and colleagues^16^. The increased use of risk‐reducing bilateral NSM may reflect growing acceptance of the procedure by physicians and patients, improvements in the cosmetic outcomes of these procedures, and increasing awareness of genetic testing and its importance in managing high‐risk patients.

The indications for risk‐reducing bilateral NSM have evolved over the past 16 years. In early reports of patients having risk‐reducing mastectomy (simple mastectomy, subcutaneous mastectomy or skin‐sparing mastectomy), a strong family history and/or a personal history of high‐risk breast lesions such as lobular carcinoma *in situ* or atypical hyperplasia were the primary indications^1^. These are similar to the indications for most patients in the present series earlier in the study. In more recent time periods, the indications have shifted to primarily those with germline genetic mutations.

Patients with *BRCA1* and *BRCA2* mutations have up to an 87 per cent lifetime risk of developing breast cancer. Most other series have focused on risk‐reducing NSM only for patients with *BRCA1* and *BRCA2* mutations, which did represent the most common indication for risk‐reducing bilateral NSM in the present series. It is noteworthy that patients with other genetic mutations associated with increased breast cancer risk (including *PTEN, TP53* and *ATM*) underwent risk‐reducing bilateral NSM in this series from 2013. Patients with *PTEN, TP53* and *ATM* have a significantly increased breast cancer risk of up to 85, 90 and 60 per cent respectively^24^, ^25^, ^26^. This series is the first to document the use of risk‐reducing bilateral NSM in patients with these mutations. Previous experience was limited to a case report^27^. No patient in the present series with a *PTEN, TP53* or *ATM* mutation developed breast cancer following risk‐reducing bilateral NSM. As multigene panel testing continues in practice, and penetrance estimates become more precise, it is anticipated that bilateral NSM for other genetic indications will become more common^28^.

Three patients in the present series had risk‐reducing bilateral mastectomy for a previous history of mantle irradiation. Patients with a history such as this for childhood cancer are known to be at increased risk of breast cancer^29^. Moskowitz and colleagues^29^ reported a cumulative incidence of breast cancer of 30 per cent by age 50 years in these patients. NSM with immediate reconstruction has been shown^30^ to be safe in these patients with a previous history of chest wall or breast irradiation. This is the first reported series of patients having risk‐reducing bilateral NSM for this indication. No patient with a prevous history of mantle irradiation in this series developed breast cancer during follow‐up.

Risk‐reducing bilateral NSM has increased over time and indications have shifted increasingly towards patients with documented genetic mutations. The present large single‐institution series supports the oncological efficacy of risk‐reducing bilateral NSM for a variety of indications, including *BRCA1/2* and other breast cancer‐associated genes that are increasingly being recognized.

## Disclosure

The authors declare no conflict of interest.

## References

[1] Hartmann LC , Schaid DJ , Woods JE , Crotty TP , Myers JL , Arnold PG *et al* Efficacy of bilateral prophylactic mastectomy in women with a family history of breast cancer. N Engl J Med 1999; 340: 77–84. 9887158 10.1056/NEJM199901143400201

[2] Pennisi VR , Capozzi A . Subcutaneous mastectomy data: a final statistical analysis of 1500 patients. Aesthetic Plast Surg 1989; 13: 15–21. 2728994 10.1007/BF01570320

[3] Meijers‐Heijboer H , van Geel B , van Putten WL , Henzen‐Logmans SC , Seynaeve C , Menke‐Pluymers MB *et al* Breast cancer after prophylactic bilateral mastectomy in women with a *BRCA1* or *BRCA2* mutation. N Engl J Med 2001; 345: 159–164. 11463009 10.1056/NEJM200107193450301

[4] Crowe JP Jr , Kim JA , Yetman R , Banbury J , Patrick RJ , Baynes D . Nipple‐sparing mastectomy: technique and results of 54 procedures. Arch Surg 2004; 139: 148–150. 14769571 10.1001/archsurg.139.2.148

[5] Crowe JP , Patrick RJ , Yetman RJ , Djohan R . Nipple‐sparing mastectomy update: one hundred forty‐nine procedures and clinical outcomes. Arch Surg 2008; 143: 1106–1110. 19015470 10.1001/archsurg.143.11.1106

[6] Orzalesi L , Casella D , Santi C , Cecconi L , Murgo R , Rinaldi S *et al* Nipple sparing mastectomy: surgical and oncological outcomes from a national multicentric registry with 913 patients (1006 cases) over a six year period. Breast 2016; 25: 75–81. 26612083 10.1016/j.breast.2015.10.010

[7] Sacchini V , Pinotti JA , Barros AC , Luini A , Pluchinotta A , Pinotti M *et al* Nipple‐sparing mastectomy for breast cancer and risk reduction: oncologic or technical problem? J Am Coll Surg 2006; 203: 704–714. 17084333 10.1016/j.jamcollsurg.2006.07.015

[8] Jensen JA , Orringer JS , Giuliano AE . Nipple‐sparing mastectomy in 99 patients with a mean follow‐up of 5 years. Ann Surg Oncol 2011; 18: 1665–1670. 21174155 10.1245/s10434-010-1475-4

[9] Yao K , Liederbach E , Tang R , Lei L , Czechura T , Sisco M *et al* Nipple‐sparing mastectomy in *BRCA1/2* mutation carriers: an interim analysis and review of the literature. Ann Surg Oncol 2015; 22: 370–376. 25023546 10.1245/s10434-014-3883-3

[10] Moo TA , Pinchinat T , Mays S , Landers A , Christos P , Alabdulkareem H *et al* Oncologic outcomes after nipple‐sparing mastectomy. Ann Surg Oncol 2016; 23: 3221–3225. 27380643 10.1245/s10434-016-5366-1

[11] Peled AW , Irwin CS , Hwang ES , Ewing CA , Alvarado M , Esserman LJ . Total skin‐sparing mastectomy in *BRCA* mutation carriers. Ann Surg Oncol 2014; 21: 37–41. 23982256 10.1245/s10434-013-3230-0

[12] Muller T , Baratte A , Bruant‐Rodier C , Bodin F , Mathelin C. Oncological safety of nipple‐sparing prophylactic mastectomy: a review of the literature on 3716 cases. Ann Chir Plast Esthet 2018; 63: e6–e13. 29030030 10.1016/j.anplas.2017.09.005

[13] Shimo A , Tsugawa K , Tsuchiya S , Yoshie R , Tsuchiya K , Uejima T *et al* Oncologic outcomes and technical considerations of nipple‐sparing mastectomies in breast cancer: experience of 425 cases from a single institution. Breast Cancer 2016; 23: 851–860. 26464007 10.1007/s12282-015-0651-6

[14] Boneti C , Yuen J , Santiago C , Diaz Z , Robertson Y , Korourian S *et al* Oncologic safety of nipple skin‐sparing or total skin‐sparing mastectomies with immediate reconstruction. J Am Coll Surg 2011; 212: 686–693. 21463813 10.1016/j.jamcollsurg.2010.12.039

[15] Manning AT , Wood C , Eaton A , Stempel M , Capko D , Pusic A *et al* Nipple‐sparing mastectomy in patients with *BRCA1/2* mutations and variants of uncertain significance. Br J Surg 2015; 102: 1354–1359. 26313374 10.1002/bjs.9884 PMC4565765

[16] Jakub JW , Peled AW , Gray RJ , Greenup RA , Kiluk JV , Sacchini V *et al* Oncologic safety of prophylactic nipple‐sparing mastectomy in a population with *BRCA* mutations: a multi‐institutional study. JAMA Surg 2018; 153: 123–129. 28903167 10.1001/jamasurg.2017.3422 PMC5838709

[17] Reynolds C , Davidson JA , Lindor NM , Glazebrook KN , Jakub JW , Degnim AC *et al* Prophylactic and therapeutic mastectomy in *BRCA* mutation carriers: can the nipple be preserved? Ann Surg Oncol 2011; 18: 3102–3109. 21947588 10.1245/s10434-011-1908-8

[18] van Verschuer VM , van Deurzen CH , Westenend PJ , Rothbarth J , Verhoef C , Luiten EJ *et al* Prophylactic nipple‐sparing mastectomy leaves more terminal duct lobular units *in situ* as compared with skin‐sparing mastectomy. Am J Surg Pathol 2014; 38: 706–712. 24698963 10.1097/PAS.0000000000000180

[19] van Verschuer VM , Maijers MC , van Deurzen CH , Koppert LB . Oncological safety of prophylactic breast surgery: skin‐sparing and nipple‐sparing *versus* total mastectomy. Gland Surg 2015; 4: 467–475. 26645001 10.3978/j.issn.2227-684X.2015.02.01 PMC4647001

[20] Maeshima Y , Oseto K , Katsuragi R , Yoshimoto Y , Takahara S , Yamauchi A . Experience with bilateral risk‐reducing mastectomy for an unaffected *BRCA* mutation carrier. J Breast Cancer 2016; 19: 218–221. 27382401 10.4048/jbc.2016.19.2.218 PMC4929266

[21] Djohan R , Gage E , Gatherwright J , Pavri S , Firouz J , Bernard S *et al* Patient satisfaction following nipple‐sparing mastectomy and immediate breast reconstruction: an 8‐year outcome study. Plast Reconstr Surg 2010; 125: 818–829. 20195110 10.1097/PRS.0b013e3181ccdaa4

[22] Wei CH , Scott AM , Price AN , Miller HC , Klassen AF , Jhanwar SM *et al* Psychosocial and sexual well‐being following nipple‐sparing mastectomy and reconstruction. Breast J 2016; 22: 10–17. 26782950 10.1111/tbj.12542 PMC4843778

[23] Romanoff A , Zabor EC , Stempel M , Sacchini V , Pusic A , Morrow M . A comparison of patient‐reported outcomes after nipple‐sparing mastectomy and conventional mastectomy with reconstruction. Ann Surg Oncol 2018; 25: 2909–2916. 29968023 10.1245/s10434-018-6585-4 PMC6205203

[24] Easton DF , Pharoah PD , Antoniou AC , Tischkowitz M , Tavtigian SV , Nathanson KL *et al* Gene‐panel sequencing and the prediction of breast‐cancer risk. N Engl J Med 2015; 372: 2243–2257. 26014596 10.1056/NEJMsr1501341 PMC4610139

[25] Tan MH , Mester JL , Ngeow J , Rybicki LA , Orloff MS , Eng C. Lifetime cancer risks in individuals with germline *PTEN* mutations. Clin Cancer Res 2012; 18: 400–407. 22252256 10.1158/1078-0432.CCR-11-2283 PMC3261579

[26] Tung N , Domchek SM , Stadler Z , Nathanson KL , Couch F , Garber JE *et al* Counselling framework for moderate‐penetrance cancer‐susceptibility mutations. Nat Rev Clin Oncol 2016; 13: 581–588. 27296296 10.1038/nrclinonc.2016.90 PMC5513673

[27] Todd J . Bi‐pedicle nipple‐sparing mastectomy (modified Letterman technique) and TIGR mesh‐assisted immediate implant reconstruction, in a patient with Cowden syndrome. Gland Surg 2016; 5: 306–311. 27294038 10.21037/gs.2015.12.01 PMC4884691

[28] Pederson HJ , Gopalakrishnan D , Noss R , Yanda C , Eng C , Grobmyer SR . Impact of multigene panel testing on surgical decision making in breast cancer patients. J Am Coll Surg 2018; 226: 560–565. 29360614 10.1016/j.jamcollsurg.2017.12.037

[29] Moskowitz CS , Chou JF , Wolden SL , Bernstein JL , Malhotra J , Novetsky Friedman D *et al* Breast cancer after chest radiation therapy for childhood cancer. J Clin Oncol 2014; 32: 2217–2223. 24752044 10.1200/JCO.2013.54.4601 PMC4100937

[30] Murphy BL , Boughey JC , Hieken TJ . Nipple‐sparing mastectomy for the management of recurrent breast cancer. Clin Breast Cancer 2017; 17: e209–e213. 28162950 10.1016/j.clbc.2016.10.011

